# Primary renal squamous cell carcinoma mimicking the renal cyst: a case report and review of the recent literature

**DOI:** 10.1186/s12894-015-0064-z

**Published:** 2015-07-23

**Authors:** Peng Jiang, Chaojun Wang, Shanwen Chen, Jun Li, Jianjian Xiang, Liping Xie

**Affiliations:** Department of Urology, The First Affiliated Hospital, School of Medicine, Zhejiang University, Qingchun Road 79, Hangzhou, 310003 Zhejiang Province China; Department of Pathology, The First Affiliated Hospital, School of Medicine, Zhejiang University, Hangzhou, Zhejiang Province China; Department of Ultrasonography, The First Affiliated Hospital, School of Medicine, Zhejiang University, Hangzhou, Zhejiang Province China

**Keywords:** Kidney, Squamous cell carcinoma

## Abstract

**Background:**

Renal squamous cell carcinoma is a rare neoplasm with poor prognosis. Chronic irritation from nephrolithiasis and/or pyelonephritis is the leading cause.

**Case presentation:**

We described a 51-year-old male patient who was admitted because of left flank pain. Ultrasonography showed a renal cyst containing calculus. However, contrast-enhanced ultrasonography and CT scan revealed an irregular-shaped mass derived from a calculi-containing cyst. Ultrasound guided biopsy confirmed the diagnosis of renal squamous cell carcinoma. The patient refused any further therapeutic management and died six months later.

**Conclusions:**

Our present case emphasizes that the careful diagnostic work-up and use of multiple imaging modalities in cases of unusual renal calculi is quite necessary, since they may carry the risk of co-existing hidden malignancy.

## Background

Squamous cell carcinoma (SCC) of the renal pelvis is a rare neoplasm, accounting only 0.5 to 0.8 % of malignant renal tumors [1]. The predisposing factors leading to development of SCC of the renal pelvis include renal calculi, infections, endogenous and exogenous chemicals, vitamin A deficiency, hormonal imbalance and radiotherapy [2–4]. We reported a case of primary SCC of the renal pelvis, which was unsuspected before biopsy, and the most recent related literatures were reviewed as well.

## Case presentation

**Fig 1:**
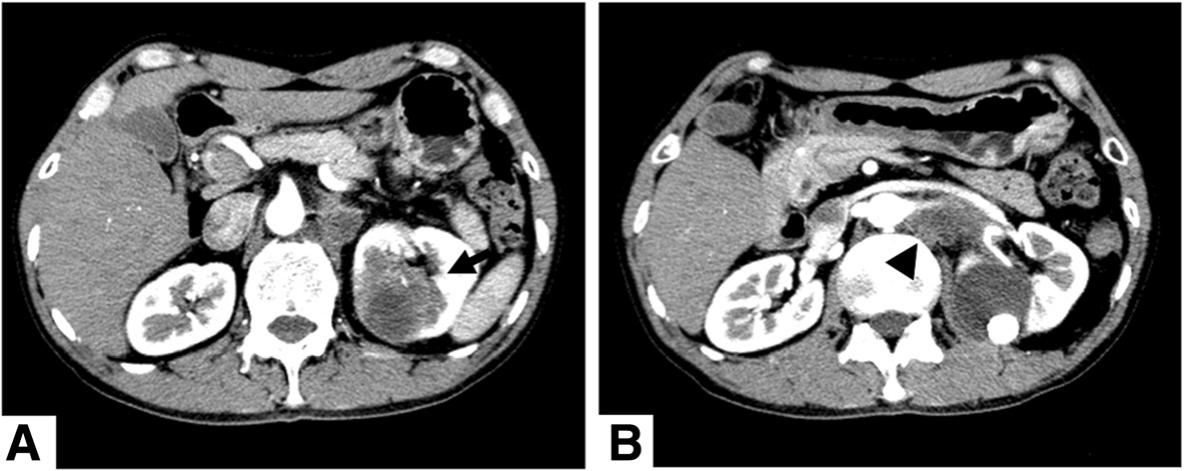
CT showed an irregular-shaped homogeneous mass (arrow) derived from the cyst and enveloped the renal pedicle

**Fig 2:**
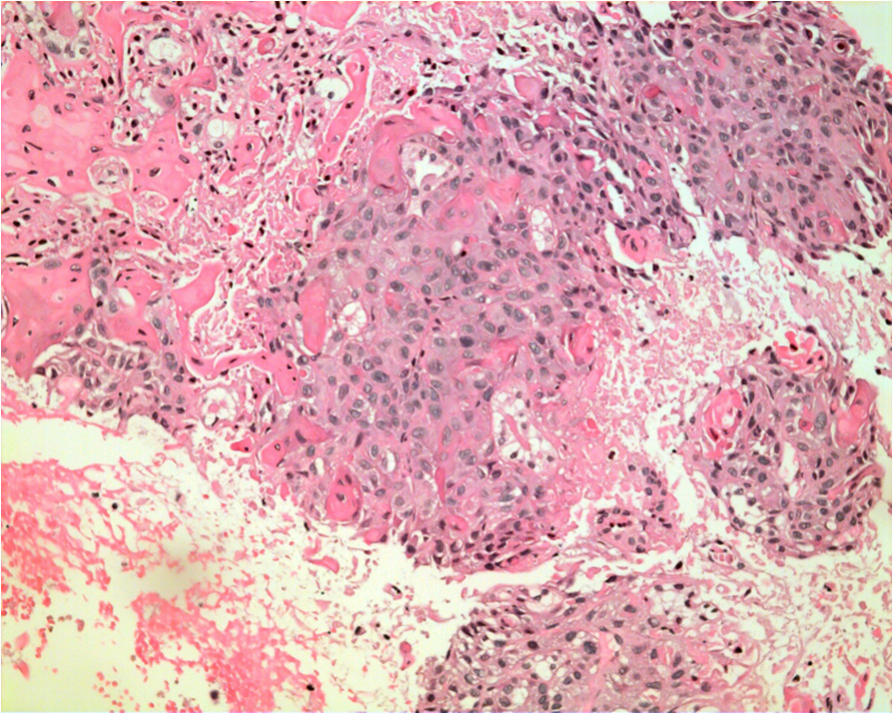
Biopsy pathology showing a high power view of squamous cell carcinoma (H&E x200)

An otherwise healthy 51-year-old male suffering from persist left flank pain for one week and was referred to the urology department. Physical examination revealed mild left costovertebral angle tenderness but was otherwise normal. Routine diagnostic work-up including chest X-ray and laboratory investigations were all within the normal range, but ultrasonography revealed a renal cyst containing calculus. Further computed tomography (CT) of the kidneys revealed an irregular-shaped homogeneous mass derived from the cyst was found. The mass enveloped the renal pedicle, aorta and inferior vena cava (Fig. 1). The mass was biopsied percutaneously under ultrasonographic guidance. The histological examination revealed squamous cell carcinoma (Fig. 2). Considering that the mass was un-resectable, the patient refused any other treatment. He returned to home hospice and unfortunately died six months later.

## Discussion

The kidney is an unusual site for SCC. Renal SCC, most of which is known to arise from collecting system, is a rare clinical entity representing only 0.5 to 0.8 % of malignant renal tumors [1]. It usually occurs in late adulthood and is reported of an equal incidence in men and women [5]. However, according to the recent literatures (Table 1), men bear a higher incidence of renal SCC, probably because of higher incidence of nephrolithiasis in men [2, 6–20]. Long-standing nephrolithiasis and/or chronic pyelonephritis are the most common causes for renal SCC. Other potential etiology have been described in the literatures, including exogenous and endogenous chemicals (e.g. arsenic), vitamin A deficiency, and prior surgery for renal stones, analgesic abuse, radiotherapy or chronic rejection in a transplant kidney [2–4]. Chronic irritation can cause squamous metaplasia of the renal collecting system, which may subsequently progress to leukoplakia and neoplasia of the urothelium, resulting in SCC of the renal pelvis. In our case, we speculated that the tumor has arisen in a chronically inflamed hydronephrotic calyx or a calyceal diverticulum with long term irritation by calculi.

**Table 1:** Characteristics of the reported cases from recent 5 years

Author	Sex	Age	Presentation	Ultrasonographic/radiological feature	Treatment	Prognosis
Bandyopadhyay et al. [6]	M	58	Heaviness and swelling in the left upper abdomen	Hydronephrosis	Nephrectomy	N/A^a^
Imriaco et al. [7]	M	69	Left flank abdominal pain	A solid mass within the left side of a horseshoe kidney, with associated large renal stones	Partial left nephrectomy	N/A
Mathur et al. [20]	M	52	Heaviness and swelling in the left upper abdomen	Non-functional kidney with dilation of renal calyces	Nephrectomy	N/A
Jain et al. [10]	M	50	Right flank pain	Staghorn calculi with right renal hydronephrosis	Nephrectomy	N/A
	M	87	Left lower abdomen pain	Left nephrolithiasis with staghorn calculi and hydronephrosis	Nephrectomy	Die in hospital because of coronary complication
	F	50	Left flank pain	Left renal and ureteric calculi with absence of corticomedullary distinction	Nephrectomy + cisplatin-based chemotherapy	Alive at 3 months after surgery
	M	53	Bilateral flank pain	Right renal calculi with hydronephrosis	Nephrectomy + cisplatin-based chemotherapy	Alive at 5 months after surgery
Paonessa et al. [11]	F	70	Vague abdominal pain	Multiple calcified areas within superior pole of the left kidney	Nephrectomy	N/A
Baseskioğlu et al. [13]	M	56	Left flank pain and fever	Hydronephrosis, staghorn calculi	Nephrectomy + radiation	Local recurrence, died 3 years later
Verma et al. [12]	M	62	Intermittent colicky pain at the right lumbar region	Right pyonephrosis with nephrolithiasis	Pyelithotomy (Palliative) + chemotherapy	N/A
Ham et al. [15]	M	69	Swelling and pain of right upper abdomen	Severe hydronephrosis with calyceal stones	Nephrectomy + Chemo	Died 7 months later
Bhaijee [14]	F	77	Weight loss and severe anemia	Left upper pole renal mass, staghorn calculus and renal vein thrombus	Nephrectomy	Asymptomatic with no evidence of recurrent or metastatic disease 6 months after surgery
Kalayci et al. [16]	M	63	10 kg weight loss	Big, non-functioning right kidney with staghorn calculi and a hypodense mass within the renal parenchyma extending to the upper pole of the right kidney	Nephrectomy	N/A
Palmer et al. [17]	F	46	Incidental finding	Large Coarse calculi with dilated renal collecting systems	Nehprectomy	Died on postoperative day 8
Wu et al. [19]	M	66	Intermittent melena, nausea, malaise, and abdominal pain	Heterogeneous renal mass containing a staghorn stone	Exploratory operation + biopsy	Died less than 5 months
Lin et al. [18]	M	56	Hematuria	Right renal staghorn calculi	Debulking surgery	Asymptomatic with no evidence of recurrent or metastatic disease 6 months after surgery
Hameed et al. [2]	F	41	Chronic backache in the right gluteal region	Complete staghorn calculus with sacral bone metastasis	Chemotherapy	Died 2 weeks after the 3rd cycle of chemotherapy

^a^N/A = Not Available

Patients with renal SCC may present with flank or abdominal pain, microscopic or gross hematuria, fever, weight loss or a palpable abdominal mass (Table 1). It could also be the incidental finding on radiographic imaging for other reasons. Establishing the diagnosis of renal SCC by imaging techniques before biopsy or surgery is a clinical dilemma. Conventional ultrasonography is the choice of imaging modality for renal diseases evaluation, but renal SCC lacks specific echoic pattern in ultrasonography. Real-time CEUS was supposed to provide additional information for improving the diagnosis [21]. CT may play a crucial role in diagnosis and staging of the tumor. The radiologic evidences of renal SCC are diverse and may appear as a solid mass with irregular shape, hydronephrosis, calcifications, or as a renal pelvic infiltrative lesion without evidence of a distinct mass. The most helpful feature in CT of renal SCC is presence of enhancing extra-luminal and exophytic mass in some cases, with an intra-luminal component [16]. Lack of specific clinical and radiologic features in renal SCC would result in diagnostic confusion. Thus, the precise histological diagnosis was usually established after nephrectomy. For the un-resectable cases, both endoscopic and percutaneous biopsy could be applied to obtain the specimen. In our case, we chose ultrasound-guided biopsy because the CT scan presented the feature of extensive peritumoral vascular invasion, which indicated that the tumor was un-resectable.

Surgical resection is regarded as the mainstay of treatment for renal SCC [18]. However, the renal SCC is aggressive in nature and concealed. Most cases usually present at an advanced stage-pT3 or higher [16]. Therefore, for the treatment of advanced disease, a multidisciplinary approach comprising of surgical treatment and adjuvant chemoradiotherapy should be applied. Still, the prognosis of renal SCC is generally poor. According to the literatures, the outcome of renal SCC is dismal with a median survival of only several months postoperatively. Holmäng *et al.* reported that the prognosis of renal SCC is usually poor with a mean survival period of 7 months [5]. The 5-year survival rate is reported less than 10 % [14]. Thus, early diagnosis, monitoring of patients with long-standing nephrolithiasis, and new treatment modalities are urgently needed to improve patients’ outcomes.

## Conclusions

For patient with unusual renal calculi, the careful diagnostic work-up with multiple imaging modalities should be applied to exclude the co-existing hidden malignancy.

## Consent

Written informed consent was obtained from the patient for publication of this case report and any accompanying images.

## Abbreviations

SCC: Squamous cell carcinoma
CEUS: Contrast-enhanced ultrasonography
CT: Computed tomography

## References

[1] Li MK, Cheung WL (1987). Squamous cell carcinoma of the renal pelvis. J Urol.

[2] Hameed ZB, Pillai SB, Hegde P, Talengala BS. Squamous cell carcinoma of the renal pelvis presenting as sacral bone metastasis. BMJ Case Rep. 2014;2014. doi:10.1136/bcr-2013-201719 10.1136/bcr-2013-201719 PMC3918639 24493112

[3] Schena S, Bogetti D, Setty S, Kadkol S, Bruno A, Testa G, Panaro F, Benedetti E, Sankary H (2004). Squamous cell carcinoma in a chronically rejected renal allograft. Am J Transplant.

[4] Papadopoulos I, Wirth B, Weichert-Jacobsen K, Loch T, Wacker HH (1996). Primary squamous cell carcinoma of the ureter and squamous adenocarcinoma of the renal pelvis: 2 case reports. J Urol.

[5] Holmang S, Lele SM, Johansson SL (2007). Squamous cell carcinoma of the renal pelvis and ureter: incidence, symptoms, treatment and outcome. J Urol.

[6] Bandyopadhyay R, Biswas S, Nag D, Ghosh AK (2010). Squamous cell carcinoma of the renal pelvis presenting as hydronephrosis. J Cancer Res Ther.

[7] Imbriaco M, Iodice D, Erra P, Terlizzi A, Di Carlo R, Di Vito C, Imbimbo C (2011). Squamous cell carcinoma within a horseshoe kidney with associated renal stones detected by computed tomography and magnetic resonance imaging. Urology.

[8] Soni HC, Jadav VJ, Sumariya B, Venkateshwaran KN, Patel N, Arya A (2012). Primary malignancy in crossed fused ectopic kidney. Abdom Imaging.

[9] Hsieh TC, Wu YC, Sun SS, Chiang IP, Yang CF, Yen KY, Kao CH (2011). Synchronous squamous cell carcinomas of the esophagus and renal pelvis. Clin Nucl Med.

[10] Jain A, Mittal D, Jindal A, Solanki R, Khatri S, Parikh A, Yadav K (2011). Incidentally detected squamous cell carcinoma of renal pelvis in patients with staghorn calculi: case series with review of the literature. ISRN Oncol.

[11] Paonessa J, Beck H, Cook S (2011). Squamous cell carcinoma of the renal pelvis associated with kidney stones: a case report. Med Oncol.

[12] Verma N, Yadav G, Dhawan N, Kumar A. Squamous cell carcinoma of kidney co-existing with renal calculi: a rare tumour. BMJ Case Rep. 2011;2011. 10.1136/bcr.10.2010.3388 PMC3063286 22707603

[13] Baseskioglu B, Yenilmez A, Acikalin M, Can C, Donmez T (2012). Verrucous carcinoma of the renal pelvis with a focus of conventional squamous cell carcinoma. Urol Int.

[14] Bhaijee F (2012). Squamous cell carcinoma of the renal pelvis. Ann Diagn Pathol.

[15] Ham BK, Kim JW, Yoon JH, Oh M, Bae JH, Park HS, du Moon G (2012). Squamous cell carcinoma must be considered in patients with long standing upper ureteral stone and pyonephrosis. Urol Res.

[16] Kalayci OT, Bozdag Z, Sonmezgoz F, Sahin N (2013). Squamous cell carcinoma of the renal pelvis associated with kidney stones: radiologic imaging features with gross and histopathological correlation. J Clin Imaging Sci.

[17] Palmer CJ, Atty C, Sekosan M, Hollowell CM, Wille MA (2014). Squamous cell carcinoma of the renal pelvis. Urology.

[18] Lin Z, Chng JK, Chong TT, Soo KC (2014). Renal pelvis squamous cell carcinoma with inferior vena cava infiltration: Case report and review of the literature. Int J Surg Case Rep.

[19] Hui Wu J, Xu Y, Qiang Xu Z, Yang K, Qiang Yang S, Shun Ma H (2014). Severe anemia and melena caused by pyeloduodenal fistula due to renal stone-associated squamous cell carcinoma. Pak J Med Sci.

[20] Mathur S, Rana P, Singh S, Goyal V, Sangwan M (2011). Incidentally detected squamous cell carcinoma in non-functioning kidney presenting as multi-cystic mass. J Surg Case Rep.

[21] Li X, Liang P, Guo M, Yu J, Yu X, Cheng Z, Han Z (2013). Real-time contrast-enhanced ultrasound in diagnosis of solid renal lesions. Discov Med.

